# Pulsed Electromagnetic Fields Disrupt Staphylococcus epidermidis Biofilms and Enhance the Antibiofilm Efficacy of Antibiotics

**DOI:** 10.1128/spectrum.01949-22

**Published:** 2022-10-31

**Authors:** Ryan B. Juncker, Beth A. Lazazzera, Fabrizio Billi

**Affiliations:** a Department of Orthopaedic Surgery, David Geffen School of Medicine at UCLA, Los Angeles, California, USA; b Department of Microbiology, Immunology, and Molecular Genetics, David Geffen School of Medicine at UCLA, Los Angeles, California, USA; University of Guelph

**Keywords:** antibiofilm, antimicrobial agents, biofilms, electromagnetic fields, hospital infections, joint infections

## Abstract

Staphylococcus epidermidis is implicated in a multitude of human infections and is one of the major causes of clinical infections in hospitals, especially at surgical sites and on indwelling medical devices, such as orthopedic implants. These infections are especially dangerous because of the S. epidermidis propensity to form biofilms, which increases resistance to antibiotics and the natural immune response. This study investigated pulsed electromagnetic fields (PEMF) as a potential treatment to combat such infections, as PEMF exposure was expected to disrupt the electrostatic forces that adhere staphylococcal cells to surfaces and to one another. To test the effect of PEMF on biofilms, S. epidermidis cultures were exposed to PEMF at various durations either during the growth phase or after a full biofilm had formed. In addition, cells were exposed to PEMF and concomitant antibiotic treatment. Biofilm viability was quantified by both crystal violet and alamarBlue assays and scanning electron microscopy. The results demonstrated that PEMF significantly inhibited biofilm formation and disrupted preformed biofilms *in vitro* while also showing synergistic biofilm inhibition when combined with antibiotics. These combined results indicate that PEMF should be considered a promising novel technique for treating S. epidermidis biofilm infections and undergo further testing *in vivo*.

**IMPORTANCE** Antibiotic resistance and biofilm infections are major issues in health care because of the lack of a successful treatment modality and poor patient outcomes. These infections are a particular issue following orthopedic surgery or trauma wherein an infection may form on an orthopedic implant or patient’s bone. The presented study demonstrates that pulsed electromagnetic fields may be a promising novel treatment for such infections and can overcome the medical challenges presented by biofilm formation. Furthermore, the effects demonstrated are even greater when combining pulsed electromagnetic field therapy with traditional antibiotics.

## INTRODUCTION

Staphylococcus epidermidis is a Gram-positive bacterium implicated in a multitude of clinical infections and brings about significant issues for the health care field. Specifically, S. epidermidis contributes to a high percentage of burn wound infections and is the leading cause of surgical-site infections (SSIs) across multiple specialties, including orthopedics and neurosurgery (1–4). S. epidermidis*-*based SSIs are a major cause of nosocomial sepsis (5) and the most frequent cause of death in postoperative hospitalized surgical patients (6). S. epidermidis is also the leading cause of medical device infections (2, 3). Specifically, S. epidermidis is a major contributor to periprosthetic joint infections (7), which are, in turn, the leading cause of revision in total knee arthroplasty (TKA) (8). Said revisions cost the health care industry over $450 million annually and are 2.5 times more expensive than revision due to aseptic loosening, the second leading cause of TKA failure (9). The current practice for a TKA revision due to periprosthetic joint infections is a two-stage procedure requiring highly invasive operations, because of the heavy debridement of the infected tissue (10–12). These revisions also have a high mortality risk resulting from related S. epidermidis*-*based sepsis. This highlights the need for an effective novel treatment, vastly improving the morbidity and mortality of countless patients undergoing TKA or other arthroplasty revision due to S. epidermidis infection.

The propensity of S. epidermidis to form biofilms, agglomerated groups of cells adhering to a surface and to one another, encased in a self-produced extracellular polymeric substance (EPS) (13, 14), is what makes it a particularly dangerous pathogen. Biofilms tend to form on surfaces such as bones and medical implants, causing infections like osteomyelitis and periprosthetic joint infections, respectively (7, 13, 14). Biofilm formation further serves to worsen S. epidermidis*-*based infections because, once fully established, small groups of aggregated cells may break off and colonize other parts of the body, spreading the infection (13). The most significant problem posed by S. epidermidis biofilm formation is its intrinsic ability to resist antibiotic treatment and the host’s natural immune response (15, 16). The primary mechanism by which biofilms resist antibiotic treatment is limiting the antibiotic’s ability to penetrate into the EPS that makes up the biofilm (17). Cells deeply encased in a biofilm also become less metabolically active, making them less susceptible to both antibiotic and immunity-based therapies. Biofilm-derived antibiotic resistance has a devastating effect on patient outcomes; Morgenstern et al. showed that biofilm-forming S. epidermidis isolates led to a 24% lower infection cure rate than non-biofilm-forming isolates (7), further highlighting the need for a novel treatment by which to combat S. epidermidis biofilm infections.

The present study investigated pulsed electromagnetic fields (PEMF) as a potential treatment for S. epidermidis biofilms. The use of PEMF is an emerging but poorly understood treatment technique throughout the medical field; it has been shown to improve bone fracture healing and is being studied as a possible treatment for cancer, rheumatoid arthritis, and osteoarthritis, among other biomedical applications (18–21). Faveri et al. showed that miniaturized electromagnetic devices inhibited polymicrobial biofilm formation on dental implants (22). Moreover, PEMF have exhibited antimicrobial effects against Gram-positive Bacillus subtilis planktonic cells (23), indicating that the effect may translate to planktonic S. epidermidis cells. This antibiofilm and antimicrobial potential, along with the noninvasive nature of PEMF, makes PEMF a strong candidate to eventually be a successful S. epidermidis biofilm infection treatment. PEMF have also been shown to stimulate the proliferation of osteoblast-like cells (24), enhance osteogenesis (25), and significantly improve wound healing (26). Thus, a PEMF-based treatment could simultaneously exhibit the intended antibiofilm effect while also stimulating bone growth and wound healing around the surgical site.

The present study tested whether the length of exposure to PEMF would (i) decrease S. epidermidis biofilm formation, (ii) decrease the viability of preformed S. epidermidis biofilm cells, and (iii) increase antibiotic efficacy in treating biofilm infections. We used an *ex vivo* approach in which S. epidermidis biofilms were grown on plastic cell culture-treated plates and exposed to various durations of PEMF through several measures of biofilm formation, including crystal violet (CV) and alamarBlue assays, and scanning electron microscopy (SEM). Through these studies, we demonstrated that PEMF have a propensity to inhibit S. epidermidis biofilms and disrupt preformed biofilms.

## RESULTS

### PEMF inhibit biofilm formation.

**FIG 1:**
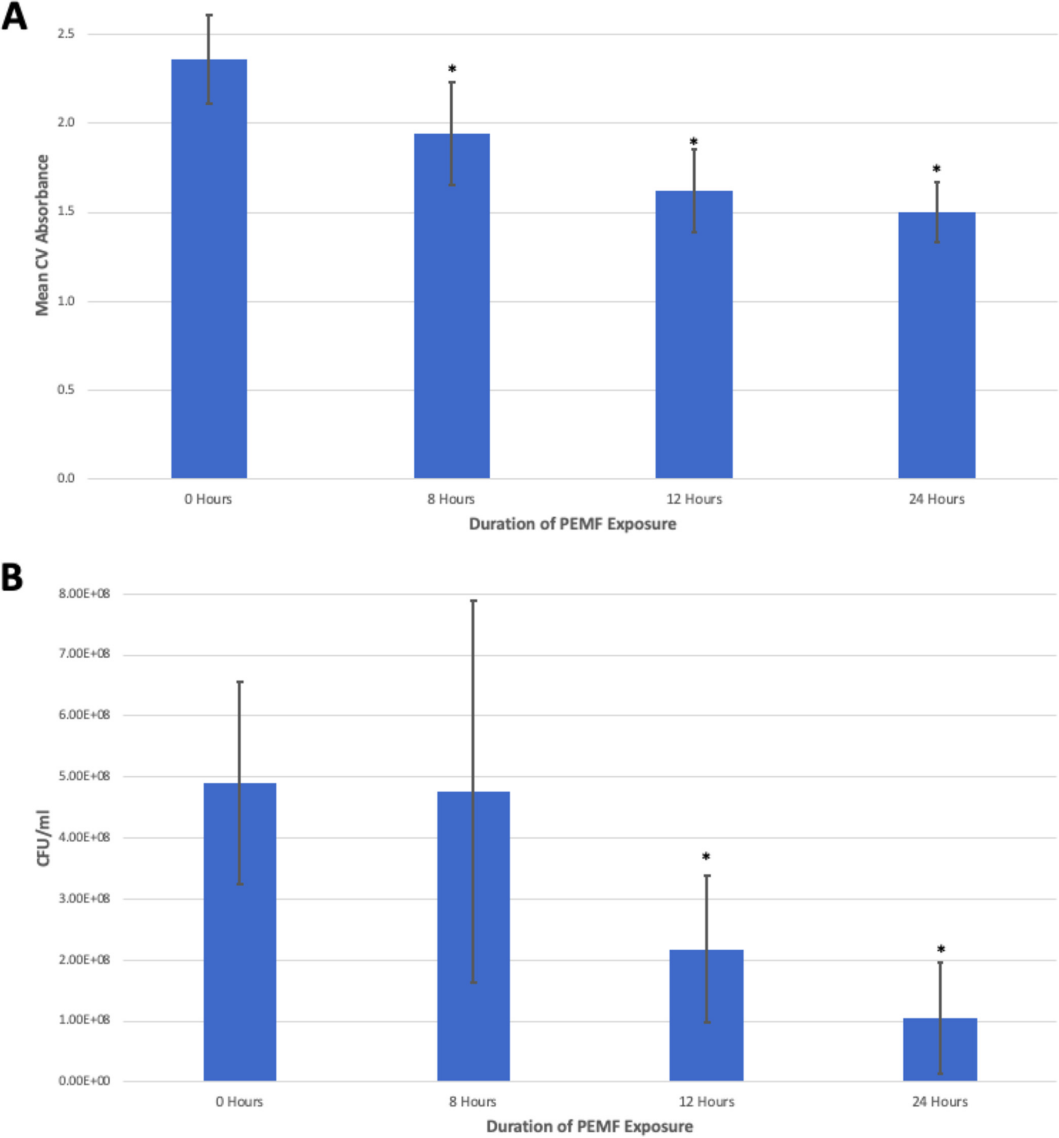
PEMF inhibit S. epidermidis biofilm formation. Plotted are the mean absorbance obtained from CV assay for total biofilm biomass (A) and CFU per milliliter of planktonic S. epidermidis cells (B). Samples were treated for the indicated length of PEMF exposure (the 8-h PEMF exposure group was exposed to PEMF for 4 h, removed from exposure for the subsequent 4 h, and then reexposed for 4 additional hours). *, statistically significant inhibition of either biofilm formation (A) or planktonic cell growth (B) compared to the no-exposure control at *P < *0.05. *n* = 36 for both panels. Error bars represent standard deviation.

The initial hypothesis tested was that PEMF would disrupt S. epidermidis surface adhesion and biofilm formation. S. epidermidis cells were inoculated into plates under conditions that support biofilm formation, and they were exposed to PEMF for various durations (4 h for 2 cycles, 12 h consecutively, or 24 h consecutively) or received no PEMF treatment. The degree of biofilm formation, quantified using a crystal violet (CV) assay, demonstrated that PEMF significantly inhibited biofilm formation (*n* = 36, *P < *0.001, *F* = 23.066, and *df *= 3) at all exposure durations, up to 35% at 24 h of exposure (*P < *0.001) (Fig. 1).

To determine whether PEMF were able to specifically disrupt biofilm formation, the impact of PEMF on planktonic cell growth was measured in CFU per milliliter. We observed a small but statistically significant overall reduction in CFU per milliliter after PEMF treatment in the 12- and 24-h exposure duration groups (*n *= 36, *P < *0.001, *F *= 8.618, and *df *= 3), which exhibited 60% (*P = *0.029) and 80% (*P = *0.001) reduction in CFU per milliliter, respectively. These data indicate that PEMF are mildly toxic to S. epidermidis cells. Due to this small effect on the viability of the planktonic cells, we cannot rule out that part of the ability of PEMF to disrupt biofilm formation is related to this inhibitory effect on growth of cells.

To confirm that the antibiofilm effect of PEMF was not due to an accumulation of heat on the polystyrene growth surface, a thermocouple was used to measure the temperature of the surface before PEMF treatment and after 8, 12, and 24 h of treatment. A negligible temperature difference was observed between the pretreatment measure (mean, 24.0°C) and 24-h treatment measure (mean, 24.3°C).

### PEMF disrupt preformed S. epidermidis biofilms.

Most infected patients do not show symptoms or undergo treatment until a biofilm has formed at the infected site. Thus, we tested the ability of PEMF to disrupt a preformed S. epidermidis biofilm. It was hypothesized that PEMF would weaken bacterial cells’ affinity to adhere to a surface and one another, breaking down the biofilm EPS and reducing total biomass. To test this hypothesis, S. epidermidis cells were grown as described above, except that the cells were allowed to grow for 24 h untreated at 37°C to form a full biofilm before being exposed to PEMF. The amount of biomass was measured using the CV assay.

**FIG 2:**
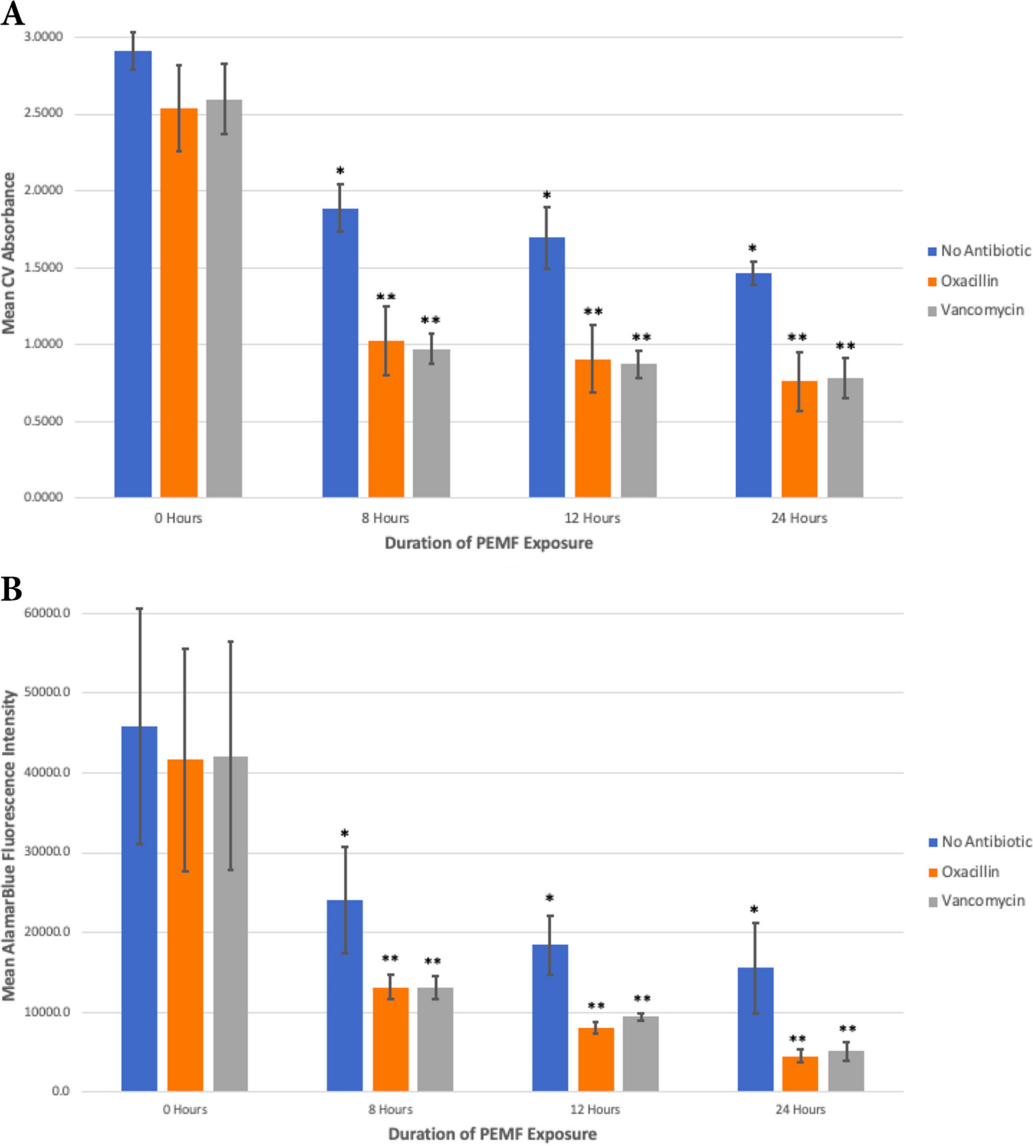
PEMF potentiate the efficacy of antibiotics disrupting preformed S. epidermidis biofilms. Plotted are the mean absorbance obtained from CV assay for total biofilm biomass (A) and the mean fluorescence intensity obtained from alamarBlue assay for cell viability (B). Samples were treated for the indicated length of PEMF exposure (the 8-h PEMF exposure group was exposed to PEMF for 4 h, removed from exposure for the subsequent 4 h, and then reexposed for 4 additional hours). In the indicated samples, oxacillin or vancomycin was added to the culture medium. *, significant disruption of biofilm compared to the no-exposure control at a *P *value of <0.001; **, significantly synergistic biofilm eradication effect of combined PEMF and antibiotic treatment compared to the theoretical additive effect (described in Materials and Methods) at a *P *value of <0.001. *n* = 54 for both panels. Error bars represent standard deviation.

As hypothesized, PEMF significantly reduced the level of preformed S. epidermidis biofilms at all three exposure durations (*P < *0.001, *n *= 48, *F *= 21.965, and *df *= 3) (Fig. 2A). The 24-h PEMF exposure group showed the largest effect, 53% biofilm reduction (*P < *0.001), compared to the sample that received no PEMF treatment. It is possible that biofilm biomass, including EPS, could have remained after PEMF treatment even if the bacterial cells in the biofilm had died. Thus, it was possible that the CV assay underestimated the impact of the PEMF on a preformed biofilm. To address this, we quantified the viability of biofilm cells using the alamarBlue assay (Fig. 2B). This showed a 66% reduction (*P < *0.001) in viable biofilm cells after 24 h of PEMF exposure. The reduction in the number of viable biofilm cells was proportional to the decrease in the amount of biofilm biomass, indicating that PEMF concomitantly disrupted biofilm components and killed biofilm cells.

### PEMF enhance the efficacy of antibiotics against S. epidermidis biofilms.

Based on the ability of PEMF to break down preformed biofilms, it was further hypothesized that PEMF would enhance the efficacy of antibiotics disrupting preformed biofilms by creating more direct access for antibiotics to S. epidermidis cells. This was tested by adding an antibiotic, oxacillin or vancomycin, to each PEMF exposure duration, using the 50% growth inhibitory concentration for each antibiotic. The amount of biofilm was again quantified by CV assay, and the amount of viable cells in the biofilm was determined by alamarBlue assay.

Neither oxacillin nor vancomycin alone significantly disrupted the S. epidermidis biofilm (Fig. 2). The largest mean theoretical additive effect of 24-h PEMF exposure and antibiotics, quantified by CV assay, was calculated for oxacillin and vancomycin to be 56% and 55% theoretical biofilm reduction, respectively. The empirically combined treatment of 24-h PEMF exposure and antibiotics proved to result in greater biofilm reduction than the theoretical additive effect (oxacillin trials, *P < *0.001, *n* = 54, *F *= 33.640, and *df *= 5; vancomycin trials, *P < *0.001, *n *= 54, *F *= 33.715, and *df *= 5). The largest mean biofilm reductions at 24 h of PEMF exposure were 72% (vancomycin) and 74% (oxacillin) as quantified by CV assay (*P < *0.001) (Fig. 2A) and 86% (vancomycin) and 89% (oxacillin) as quantified by alamarBlue assay (*P < *0.001) (Fig. 2B). These data indicate that PEMF and antibiotics act synergistically.

**FIG 3:**
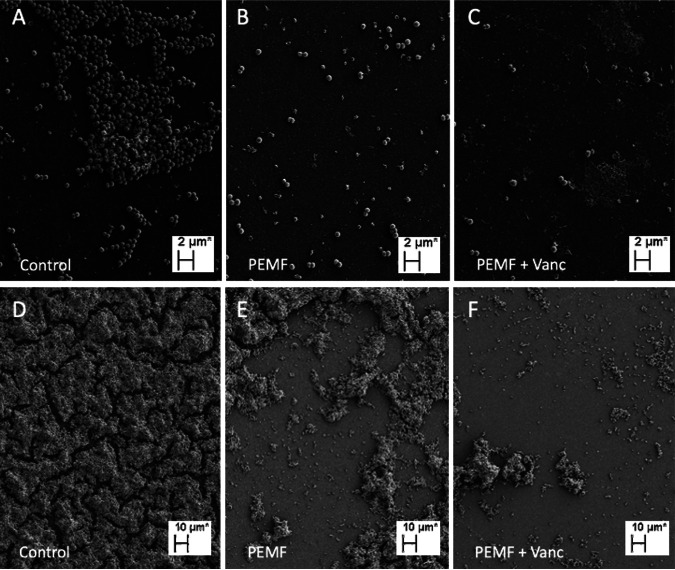
PEMF reduce the number of surface-adhered S. epidermidis biofilm cells. Shown are SEM images of S. epidermidis grown on a cell culture-treated, polystyrene, 24-well plate. (A to C) S. epidermidis ATCC 14990; (D to F) S. epidermidis ATCC 35984. Panels A and D show the control, which was S. epidermidis that was not exposed to PEMF or vancomycin. Panels B and E show S. epidermidis after 24 h of PEMF exposure. Panels C and F show S. epidermidis after 24 h of PEMF exposure and simultaneous vancomycin treatment.

To assess what impact PEMF alone and in combination with antibiotics had on the biofilm structure, the biofilms were analyzed through scanning electron microscopy (SEM) imaging (Fig. 3). In the absence of PEMF, we observed large clusters of cells adhered to the surface for S. epidermidis strain 14990, the strain used in all the previous experiments (Fig. 3A). After treatment with PEMF or PEMF and vancomycin, only individual cells adhered to the surface and no biofilm was observed (Fig. 3B and C). We also assayed S. epidermidis strain ATCC 35984 by SEM. This strain showed a thick biofilm attached to the surface (Fig. 3D). After treatment with PEMF, there was a reduction in the amount of biofilm attached the surface. Addition of vancomycin to PEMF treatment resulted in a further reduction of the amount of biofilm. These results are consistent with the indication that PEMF can disrupt interactions between cells and/or between cells and the surface.

## DISCUSSION

S. epidermidis remains a major contributor to many devastating clinical infections, particularly surgical-site infections and orthopedic implant infections, such as periprosthetic joint infections and spinal implant infections (7, 27). Biofilm formation significantly increases the morbidity and mortality of such infections by increasing bacterial resistance to antibiotics and evasion of the host immune response (7, 13, 14). As there is currently no FDA-approved treatment for biofilm infections, a novel solution would provide massive value to surgeons and patients alike. This study successfully demonstrated a novel approach to treating such infections, using PEMF to disrupt the biofilm and to work synergistically with multiple antibiotics.

This study reveals that not only can PEMF inhibit S. epidermidis biofilm formation and disrupt preformed biofilms, but also, when combined with an antibiotic, it can eradicate up to 89% of an established staphylococcal biofilm. PEMF made the antibiotics significantly more active on the biofilm cells than they were on their own. The ability of PEMF to disrupt existing biofilms carries much more weight toward an eventual clinical solution than the ability to inhibit the formation of a biofilm, as clinical infections will more than likely have already formed a full biofilm by the time a patient presents with symptoms. Much of the importance of this novel approach lies in its noninvasive nature, which, if integrated clinically, would greatly decrease both cost and patient discomfort compared to the current two-stage revision technique for periprosthetic joint infections (10, 11). Similar success has been shown for alternating magnetic field therapy by Chopra et al. in 2017 (28) and Wang et al. in 2021 (29); however, important differences are notable. Both previous studies used an alternating magnetic field to eradicate biofilms by generating heat in a metal implant, essentially burning the bacteria (28, 29). While successful, the heat produced may also affect surrounding tissue and be detrimental to the osseointegration of an orthopedic implant. On the other hand, the presented study produced similar eradication effects while using a lower frequency for the PEMF signal to avoid any significant concerns about heat being produced and damaging tissue. For comparison, Chopra et al. and Wang et al. utilized 500-kHz and >100-kHz magnetic fields, respectively, while this study used a 40-kHz field. While it is possible that heat plays a role in the effect of 40-kHz frequency PEMF on metal surfaces, we confirmed that the heat production by PEMF on polystyrene surfaces in this study was negligible.

The mechanism by which PEMF disrupt S. epidermidis biofilms is currently unknown. However, having treated biofilms grown on polystyrene in the presented study, in contrast to the aforementioned studies by Chopra et al. and Wang et al., which used metal surfaces, suggests that PEMF have a S. epidermidis biofilm disruption mechanism independent of heat conduction through metal. We suspect that much of the effect is due to prolonged PEMF exposure disrupting electrostatic interactions between bacteria and the growth surface and between adjacent staphylococcal cells. Additionally, PEMF may induce an electroporation effect due to the induction of eddy current flow through the biofilm sample, weakening the cell membrane and creating pores in the membrane or biofilm EPS and making the biofilm inefficient at protecting against antibiotics and the immune response. Electrostatic interactions have been shown to play a major role in biofilm attachment and strength across both Gram-positive and Gram-negative biofilm-forming species (30–33). The direct antimicrobial effect of PEMF on S. epidermidis cells, likely due to induction of increased permeabilization and local magnetic hyperthermia (23), may additionally contribute to their biofilm disruption mechanism. SEM images in Fig. 3 provide further support, showing that PEMF exposure drastically reduces the adhered S. epidermidis cells. Further, much of biofilm-based antibiotic resistance is caused by the biofilm matrix and structure essentially blocking antibiotics’ access to bacterial cells, physically limiting their capability to affect bacteria causing infection (15–17). Thus, the synergistic effect of PEMF and antibiotics was likely due to weakened electrostatic forces in the biofilm matrix, resulting in opening of pores and/or weakening the structure enough to give antibiotics direct access to S. epidermidis cells. Consistent with this, we observed by SEM that PEMF treatment reduced the size of the biofilm into smaller colonies or individual cells, likely allowing for improved antibiotic access to cells.

With such promising results, the limitations of this study are important to note. The study was performed using only cell culture-treated polystyrene plates, limiting its direct generalizability to the various metal alloys used in orthopedic implants. However, because cell culture-treated plates were used, and it has been well established that staphylococcal biofilms grow well on multiple plastic and metal surfaces, it was assumed that this model well represented PEMF interaction with biofilm on a variety of surfaces. Additionally, only two strains of S. epidermidis were tested in the presented study. While it is encouraging that PEMF and antibiotic treatment showed similar results with both strains, it will be important to test this treatment on other clinically relevant bacteria, such as Staphylococcus aureus and Pseudomonas aeruginosa, along with multispecies biofilms. Finally, specific limitations exist with any *ex vivo* infection model, as both biofilm growth and PEMF will presumably act differently when exposed to a flow state and natural immune factors in human or animal blood. While such challenges must be addressed en route to any *in vivo* or clinical studies, the presented data still makes a strong case for the potential of PEMF as an antibiofilm therapy.

Despite these limitations, we believe that the observed data establish PEMF as a promising method by which to prevent and disrupt staphylococcal biofilm infections. PEMF not only significantly inhibited S. epidermidis biofilm growth but also radically reduced preformed biofilms. Moreover, when oxacillin or vancomycin was simultaneously administered at a concentration that inhibited 50% of growth, the data showed that PEMF synergistically enhanced the biofilm disruption ability of both antibiotics, further supporting the ability of PEMF to inhibit S. epidermidis biofilm strength and structure.

A clinical therapy derived from this technique would be a major improvement over the current two-stage revision process for periprosthetic joint infections for surgeons and patients alike. The ability of PEMF to act synergistically in conjunction with antibiotics suggests their potential to do the same with other antimicrobial and antibiofilm therapies, such as magnetic nanoparticles, synthetic antibodies, antimicrobial peptides, and a multitude of other therapies currently being tested, cementing their importance for inclusion in future research in the field.

## MATERIALS AND METHODS

### Materials.

All experiments were performed on S. epidermidis strain ATCC 14990, unless otherwise indicated, and then S. epidermidis strain ATCC 35984 was used, which is known to be a particularly strong biofilm former (34). Trypticase soy broth (TSB) (30 g L^−1^ in water, autoclaved at 121°C for 15 min; BD Bacto TSB from Fisher Scientific) was used for *in vitro* growth of bacterial cell cultures, and glucose was added to the medium at a final concentration of 0.25% when indicated. Sterile Falcon 6-well, clear, flat-bottom, tissue culture-treated cell culture plates (Corning; lot 1059012) were used for crystal violet assays, and sterile CELLSTAR 24-well, polystyrene, clear, tissue culture-treated cell culture plates (Greiner; lot E100501A) were used for alamarBlue assays.

Crystal violet (CV) stain (0.41% [wt/vol] in 12% ethanol; Thermo Fisher Scientific; lot V70290) and alamarBlue cell viability reagent (Invitrogen; lot 2263437) (used as 5.0% alamarBlue in TSB) were used for biofilm growth and viability quantification, respectively, both of which are established measures of S. epidermidis biofilm viability (35, 36). Wash buffer was used to rinse medium and planktonic S. epidermidis cells from all plated wells to ensure that only biofilm-adhered cells remained before quantification (37).

Oxacillin (Sigma-Aldrich; source 1b190146, batch 0000089022) and vancomycin (Sigma-Aldrich; lot 048M4087V) were the two antibiotics tested in conjunction with PEMF.

The PEMF apparatus was custom-made in our laboratory and was previously described by Ghalayani Esfahani et al. (35). Briefly, the PEMF apparatus consisted of a custom-made acrylic shelved rack with wraparound Helmholtz coils, a signal generator (KEYSIGHT 33220A function/arbitrary waveform generator, 20 MHz), and a power supply (GwinSTEK GPS-18500 laboratory DC power supply) generating 15 V of current, inducing an estimate electric field on the sample of 0.00015 mV. The signal had a characteristic frequency of 40 kHz and was characterized by a square waveform for all trials. The intensity of the magnetic field was measured at various points on the rack with a Gaussian probe, and the cell culture plates were positioned where the field had a constant intensity. A digital oscilloscope (TBS1000C; Tektronix, Beaverton, OR) was used to monitor the signal waveform. The effect of PEMF on S. epidermidis biofilms was tested by exposing bacterial samples to PEMF for various durations (4 h for 2 cycles, 12 h consecutively, or 24 h consecutively). All plates not under PEMF treatment were kept in a 37°C incubator; this temperature is identical to that of the PEMF incubator.

### Biofilm growth.

S. epidermidis biofilms were grown by initially incubating freezer stock S. epidermidis cell scrapings in TSB medium at 37°C for 24 h with shaking. Subsequently, the cells were diluted 1:100 into TSB media containing 0.25% glucose and then aliquoted, in 3-mL or 1-mL aliquots, into 6-well or 24-well plates, respectively. To test the ability of PEMF to disrupt preformed biofilms, inoculated plates were incubated for 24 h to allow biofilm formation to occur. Negative controls of only TSB medium containing 0.25% glucose (i.e., no cells) were also included for all plates to ensure that no contamination occurred.

### Biofilm quantification.

The crystal violet (CV) assay was utilized to quantify biofilm formation as previously described (35). After incubation of the biofilm in the plates with or without treatment, the media and any nonadhered cells were removed. The wells of the plates were then washed with wash buffer three times to ensure that only biofilm-adhered cells remained. Next, wells were incubated with CV at room temperature for 15 min. The CV stain was then removed, and wells were washed twice with sterile water. An 80% ethanol–20% acetone solution was then added and incubated for 20 min at room temperature to solubilize the dye. Absorbance readings were thereafter obtained at 570 nm and recorded for each well (BMG LABTECH; FLUOstar Omega plate reader).

alamarBlue was utilized to measure the viability of biofilm cells. The alamarBlue assay was performed by removing the growth media and washing all wells with wash buffer as described above for the CV assay. A solution of 5% alamarBlue in TSB medium containing 0.25% glucose was next added to the wells, and fluorescence readings were obtained after 30 min. For this assay, the FLUOstar Omega plate reader, mentioned above, was set to well-scanning, bottom reading optic mode. The gain was set at 1,100, and fluorescence was read at 544 and 590 nm (excitation and emission). Samples underwent 10 s of shaking at 100 rpm directly prior to each reading.

### Planktonic cell quantification.

The value for CFU per milliliter was used as the number of planktonic S. epidermidis cells posttreatment. Planktonic cells were first diluted serially, and 20-μL aliquots from the 10^−2^ to 10^−5^ dilutions were then spread on Luria broth (LB) plates and allowed to grow overnight before CFU counts were taken the following day. CFU per milliliter were then calculated for each using the following equation: CFU per milliliter = (number of colonies × dilution factor)/volume.

### Determination of antibiotic concentrations to inhibit 50% growth.

To determine the effect of PEMF exposure on antibiotic treatment, the concentrations of both oxacillin and vancomycin that would inhibit 50% growth were determined for the specific S. epidermidis strain used. S. epidermidis were first incubated in TSB medium at 37°C for 24 h with shaking. The culture was subsequently diluted 1:100 into TSB medium containing 0.25% glucose, and 100-μL aliquots of the diluted cells were added to 96-well cell culture plates. The antibiotic being tested was then serially diluted down the plate, resulting in 12 final concentrations ranging from 500 μg/mL to 0.25 μg/mL. After 24 h, readings of the optical density at 600 nm (OD_600_) of the wells were taken. The lowest concentration of antibiotic to show ≥50% inhibition of planktonic S. epidermidis cell growth was quantified by OD_600_ readings. The 50% inhibition concentrations of oxacillin and vancomycin were 1.5 μL/mL and 4.0 μL/mL, respectively.

### Biofilm fixation and SEM imaging.

The structures of S. epidermidis biofilms were determined using scanning electron microscopy (SEM). Biofilms were grown and treated on tissue culture-treated plastic slides cut from 24-well plates and placed within the 24-well cell culture plates before bacterial cultures were inoculated. These slides were cut so SEM imaging could be performed on a surface equivalent to that used in all quantitative experiments without disturbing the biofilm sample during the imaging process. Fixation was performed by initially washing all slides twice in phosphate-buffered saline (PBS) and then incubating them in 2.5% glutaraldehyde for 48 h. At the conclusion of the 48-h fixation period, slides were rinsed in PBS again for two cycles of 10 min each. This step was followed by a 1-h postfixation step in 2% osmium tetroxide (OsO_4_). The samples were then dehydrated using an ascending ethanol series in which samples were incubated in 25%, 50%, 75%, and then, finally, 97% ethanol for 30 min each. All samples were then dried overnight, sputter coated, and imaged using variable-pressure SEM.

### Reproducibility and statistical analyses.

To confirm the reproducibility of results, all experiments were run identically at least three times before results were analyzed and reported. All groups were compared using 1-way analysis of variance (ANOVA) with Tukey *post hoc* tests on SPSS statistics, with statistical significance defined as a *P *value of *≤*0.05. One major comparison of interest was the theoretical additive effect of PEMF and antibiotic treatment versus the empirical effect of simultaneous PEMF and antibiotic treatment. The theoretical additive effect was calculated using a simple probability calculation equating theoretical percent biofilm reduction to *P*(*A*) + [1 − *P*(*A*)] × *P*(*B*); *A* represents the percent biofilm reduction by PEMF alone and *B* represents reduction by antibiotic alone. Importantly, this effect was calculated individually for each replicate to allow a true statistical comparison to be performed (via 1-way ANOVA plus Tukey *post hoc* tests as described above). The data that were generated from these calculations were compared to the empirical data collected from simultaneous PEMF and antibiotic treatment experimental groups to determine if statistically significant synergism had occurred.

## Supplementary Material

Reviewer comments

## References

[1] Hubab M, Maab H, Hayat A, Ur Rehman M. 2020. Burn wound microbiology and the antibiotic susceptibility patterns of bacterial isolates in three burn units of Abbottabad, Pakistan. J Burn Care Res 41:1207–1211. doi:10.1093/jbcr/iraa073. 32403126

[2] Oliveira WF, Silva PMS, Silva RCS, Silva GMM, Machado G, Coelho L, Correia MTS. 2018. Staphylococcus aureus and Staphylococcus epidermidis infections on implants. J Hosp Infect 98:111–117. doi:10.1016/j.jhin.2017.11.008. 29175074

[3] Perez K, Patel R. 2018. Survival of Staphylococcus epidermidis in fibroblasts and osteoblasts. Infect Immun 86:e00237-18. doi:10.1128/IAI.00237-18. 30061380 PMC6204734

[4] López Pereira P, Díaz-Agero Pérez C, López Fresneña N, Las Heras Mosteiro J, Palancar Cabrera A, Rincón Carlavilla ÁL, Aranaz Andrés JM. 2017. Epidemiology of surgical site infection in a neurosurgery department. Br J Neurosurg 31:10–15. doi:10.1080/02688697.2016.1260687. 27905216

[5] Oliveira F, Lima T, Correia A, Silva AM, Soares C, Morais S, Weißelberg S, Vilanova M, Rohde H, Cerca N. 2022. Involvement of the iron-regulated loci *hts* and *fhuC* in biofilm formation and survival of Staphylococcus epidermidis within the host. Microbiol Spectr 10:e02168-21. doi:10.1128/spectrum.02168-21. 35019768 PMC8754135

[6] Otto M. 2017. Staphylococcus epidermidis: a major player in bacterial sepsis? Future Microbiol 12:1031–1033. doi:10.2217/fmb-2017-0143. 28748707 PMC5627029

[7] Morgenstern M, Post V, Erichsen C, Hungerer S, Bühren V, Militz M, Richards RG, Moriarty TF. 2016. Biofilm formation increases treatment failure in Staphylococcus epidermidis device-related osteomyelitis of the lower extremity in human patients. J Orthop Res 34:1905–1913. doi:10.1002/jor.23218. 26925869

[8] Koh CK, Zeng I, Ravi S, Zhu M, Vince KG, Young SW. 2017. Periprosthetic joint infection is the main cause of failure for modern knee arthroplasty: an analysis of 11,134 knees. Clin Orthop Relat Res 475:2194–2201. doi:10.1007/s11999-017-5396-4. 28573549 PMC5539036

[9] Okafor C, Hodgkinson B, Nghiem S, Vertullo C, Byrnes J. 2021. Cost of septic and aseptic revision total knee arthroplasty: a systematic review. BMC Musculoskelet Disord 22:706. doi:10.1186/s12891-021-04597-8. 34407779 PMC8371784

[10] Ludwick L, Chisari E, Wang J, Clarkson S, Collins L, Parvizi J. 2021. Emergence of antibiotic resistance across two-stage revision for periprosthetic joint infection. J Arthroplasty 36:2946–2950. doi:10.1016/j.arth.2021.04.007. 33934949

[11] Lenguerrand E, Whitehouse MR, Beswick AD, Kunutsor SK, Foguet P, Porter M, Blom AW. 2019. Risk factors associated with revision for prosthetic joint infection following knee replacement: an observational cohort study from England and Wales. Lancet Infect Dis 19:589–600. doi:10.1016/S1473-3099(18)30755-2. 31005559 PMC6531378

[12] Gehrke T, Alijanipour P, Parvizi J. 2015. The management of an infected total knee arthroplasty. Bone Joint J 97-B(Suppl A):20–29. doi:10.1302/0301-620X.97B10.36475. 26430083

[13] Paharik AE, Horswill AR. 2016. The staphylococcal biofilm: adhesins, regulation, and host response. Microbiol Spectr 4:4.2.06. doi:10.1128/microbiolspec.VMBF-0022-2015. PMC4887152 27227309

[14] Takahashi C, Sato M, Sato C. 2021. Biofilm formation of Staphylococcus epidermidis imaged using atmospheric scanning electron microscopy. Anal Bioanal Chem 413:7549–7558. doi:10.1007/s00216-021-03720-x. 34671824

[15] Schilcher K, Horswill AR. 2020. Staphylococcal biofilm development: structure, regulation, and treatment strategies. Microbiol Mol Biol Rev 84:e00026-19. doi:10.1128/MMBR.00026-19. 32792334 PMC7430342

[16] Ortega-Peña S, Martínez-García S, Rodríguez-Martínez S, Cancino-Diaz ME, Cancino-Diaz JC. 2020. Overview of Staphylococcus epidermidis cell wall-anchored proteins: potential targets to inhibit biofilm formation. Mol Biol Rep 47:771–784. doi:10.1007/s11033-019-05139-1. 31642039

[17] Hall CW, Mah TF. 2017. Molecular mechanisms of biofilm-based antibiotic resistance and tolerance in pathogenic bacteria. FEMS Microbiol Rev 41:276–301. doi:10.1093/femsre/fux010. 28369412

[18] Vadalà M, Morales-Medina JC, Vallelunga A, Palmieri B, Laurino C, Iannitti T. 2016. Mechanisms and therapeutic effectiveness of pulsed electromagnetic field therapy in oncology. Cancer Med 5:3128–3139. doi:10.1002/cam4.861. 27748048 PMC5119968

[19] Ross CL, Ang DC, Almeida-Porada G. 2019. Targeting mesenchymal stromal cells/pericytes (MSCs) with pulsed electromagnetic field (PEMF) has the potential to treat rheumatoid arthritis. Front Immunol 10:266. doi:10.3389/fimmu.2019.00266. 30886614 PMC6409305

[20] Yang X, He H, Ye W, Perry TA, He C. 2020. Effects of pulsed electromagnetic field therapy on pain, stiffness, physical function, and quality of life in patients with osteoarthritis: a systematic review and meta-analysis of randomized placebo-controlled trials. Phys Ther 100:1118–1131. doi:10.1093/ptj/pzaa054. 32251502

[21] Mohajerani H, Tabeie F, Vossoughi F, Jafari E, Assadi M. 2019. Effect of pulsed electromagnetic field on mandibular fracture healing: a randomized control trial, (RCT). J Stomatol Oral Maxillofac Surg 120:390–396. doi:10.1016/j.jormas.2019.02.022. 30836195

[22] Faveri M, Miquelleto DEC, Bueno-Silva B, Pingueiro JMS, Figueiredo LC, Dolkart O, Yakobson E, Barak S, Feres M, Shibli JA. 2020. Antimicrobial effects of a pulsed electromagnetic field: an in vitro polymicrobial periodontal subgingival biofilm model. Biofouling 36:862–869. doi:10.1080/08927014.2020.1825694. 32993357

[23] Novickij V, Stanevičienė R, Vepštaitė-Monstavičė I, Gruškienė R, Krivorotova T, Sereikaitė J, Novickij J, Servienė E. 2017. Overcoming antimicrobial resistance in bacteria using bioactive magnetic nanoparticles and pulsed electromagnetic fields. Front Microbiol 8:2678. doi:10.3389/fmicb.2017.02678. 29375537 PMC5767227

[24] Miyamoto H, Sawaji Y, Iwaki T, Masaoka T, Fukada E, Date M, Yamamoto K. 2019. Intermittent pulsed electromagnetic field stimulation activates the mTOR pathway and stimulates the proliferation of osteoblast-like cells. Bioelectromagnetics 40:412–421. doi:10.1002/bem.22207. 31338867

[25] Benya PD, Kavanaugh A, Zakarian M, Söderlind P, Jashashvili T, Zhang N, Waldorff EI, Ryaby JT, Billi F. 2021. Pulsed electromagnetic field (PEMF) transiently stimulates the rate of mineralization in a 3-dimensional ring culture model of osteogenesis. PLoS One 16:e0244223. doi:10.1371/journal.pone.0244223. 33539401 PMC7861434

[26] Patiño O, Grana D, Bolgiani A, Prezzavento G, Miño J, Merlo A, Benaim F. 1996. Pulsed electromagnetic fields in experimental cutaneous wound healing in rats. J Burn Care Rehabil 17:528–531. doi:10.1097/00004630-199611000-00009. 8951540

[27] Karau MJ, Zhang C, Mandrekar JN, Kohrs NJ, Puleo DA, van Wijnen AJ, Patel R, Boyce TG, Larson AN, Milbrandt TA. 2020. Topical vancomycin for treatment of methicillin-resistant Staphylococcus epidermidis infection in a rat spinal implant model. Spine Deform 8:553–559. doi:10.1007/s43390-020-00087-4. 32078142

[28] Chopra R, Shaikh S, Chatzinoff Y, Munaweera I, Cheng B, Daly SM, Xi Y, Bing C, Burns D, Greenberg DE. 2017. Employing high-frequency alternating magnetic fields for the non-invasive treatment of prosthetic joint infections. Sci Rep 7:7520. doi:10.1038/s41598-017-07321-6. 28790407 PMC5548742

[29] Wang Q, Vachon J, Prasad B, Pybus CA, Lapin N, Chopra R, Greenberg DE. 2021. Alternating magnetic fields and antibiotics eradicate biofilm on metal in a synergistic fashion. NPJ Biofilms Microbiomes 7:68. doi:10.1038/s41522-021-00239-y. 34385452 PMC8360946

[30] Müller C, Lüders A, Hoth-Hannig W, Hannig M, Ziegler C. 2010. Initial bioadhesion on dental materials as a function of contact time, pH, surface wettability, and isoelectric point. Langmuir 26:4136–4141. doi:10.1021/la903299y. 19888741

[31] Hartvig RA, van de Weert M, Østergaard J, Jorgensen L, Jensen H. 2011. Protein adsorption at charged surfaces: the role of electrostatic interactions and interfacial charge regulation. Langmuir 27:2634–2643. doi:10.1021/la104720n. 21322572

[32] Petrova OE, Sauer K. 2012. Sticky situations: key components that control bacterial surface attachment. J Bacteriol 194:2413–2425. doi:10.1128/JB.00003-12. 22389478 PMC3347170

[33] Nakanishi EY, Palacios JH, Godbout S, Fournel S. 2021. Interaction between biofilm formation, surface material and cleanability considering different materials used in pig facilities—an overview. Sustainability 13:5836. doi:10.3390/su13115836.

[34] Okajima Y, Kobayakawa S, Tsuji A, Tochikubo T. 2006. Biofilm formation by Staphylococcus epidermidis on intraocular lens material. Invest Ophthalmol Vis Sci 47:2971–2975. doi:10.1167/iovs.05-1172. 16799041

[35] Ghalayani Esfahani A, Lazazzera B, Draghi L, Farè S, Chiesa R, De Nardo L, Billi F. 2019. Bactericidal activity of gallium-doped chitosan coatings against staphylococcal infection. J Appl Microbiol 126:87–101. doi:10.1111/jam.14133. 30329212

[36] Pettit RK, Weber CA, Kean MJ, Hoffmann H, Pettit GR, Tan R, Franks KS, Horton ML. 2005. Microplate Alamar blue assay for Staphylococcus epidermidis biofilm susceptibility testing. Antimicrob Agents Chemother 49:2612–2617. doi:10.1128/AAC.49.7.2612-2617.2005. 15980327 PMC1168683

[37] Hamon MA, Lazazzera BA. 2001. The sporulation transcription factor Spo0A is required for biofilm development in Bacillus subtilis. Mol Microbiol 42:1199–1209. doi:10.1046/j.1365-2958.2001.02709.x. 11886552

