# Supplementary material for: Pulsed Electromagnetic Fields Disrupt Staphylococcus epidermidis Biofilms and Enhance the Antibiofilm Efficacy of Antibiotics
Source: Microbiol Spectr. 2022 Oct 31;10(6):e01949-22. doi: 10.1128/spectrum.01949-22 (PMC9769884; doi:10.1128/spectrum.01949-22)
Supplement: Reviewer comments [file reviewer-comments.pdf]

AMERICAN SOCIETY FOR MICROBIOLOGY | Microbiology Spectrum

Pulsed Electromagnetic Fields Disrupt Staphylococcus epidermidis Biofilms and Enhance the Anti-Biofilm Efficacy of Antibiotics

Ryan Juncker, Beth Lazazzera, and Fabrizio Billi

Corresponding Author(s): Ryan Juncker, UCLA David Geffen School of Medicine

Review Timeline:

Submission Date:	September 15, 2022
Accepted:	September 24, 2022

Editor: Cezar Khursigara

Reviewer(s): The reviewers have opted to remain anonymous.

Transaction Report:

(Note: With the exception of the correction of typographical or spelling errors that could be a source of ambiguity, letters and reports are not edited. The original formatting of letters and referee reports may not be reflected in this compilation.)

DOI: https://doi.org/10.1128/spectrum.01949-22

September 24, 2022

Dr. Ryan B Juncker
UCLA David Geffen School of Medicine
Orthopaedic Surgery
615 Charles E Young Dr. S
Rm 410
Los Angeles, California 90095

Re: Spectrum01949-22 (Pulsed Electromagnetic Fields Disrupt Staphylococcus epidermidis Biofilms and Enhance the Anti-Biofilm Efficacy of Antibiotics)

Dear Dr. Ryan B Juncker:

Your manuscript has been accepted, and I am forwarding it to the ASM Journals Department for publication. You will be notified when your proofs are ready to be viewed.

The ASM Journals program strives for constant improvement in our submission and publication process. Please tell us how we can improve your experience by taking this quick Author Survey.

As an open-access publication, Spectrum receives no financial support from paid subscriptions and depends on authors' prompt payment of publication fees as soon as their articles are accepted. You will be contacted separately about payment when the proofs are issued; please follow the instructions in that e-mail. Arrangements for payment must be made before your article is published. For a complete list of **Publication Fees**, including supplemental material costs, please visit ourwebsite.

ASM policy requires that data be available to the public upon online posting of the article, so please verify all links to sequence records, if present, and make sure that each number retrieves the full record of the data. If a new accession number is not linked or a link is broken, provide production staff with the correct URL for the record. If the accession numbers for new data are not publicly accessible before the expected online posting of the article, publication of your article may be delayed; please contact the ASM production staff immediately with the expected release date.

Corresponding authors may join or renew ASM membership to obtain discounts on publication fees. Need to upgrade your membership level? Please contact Customer Service at Service@asmusa.org.

Thank you for submitting your paper to Spectrum.

Sincerely,

Cezar Khursigara
Editor, Microbiology Spectrum

Journals Department
American Society for Microbiology
1752 N St., NW
Washington, DC 20036
E-mail: spectrum@asmusa.org
